# Urothelial Defects from Targeted Inactivation of Exocyst Sec10 in Mice Cause Ureteropelvic Junction Obstructions

**DOI:** 10.1371/journal.pone.0129346

**Published:** 2015-06-05

**Authors:** Ben Fogelgren, Noemi Polgar, Vanessa H. Lui, Amanda J. Lee, Kadee-Kalia A. Tamashiro, Josephine Andrea Napoli, Chad B. Walton, Xiaofeng Zuo, Joshua H. Lipschutz

**Affiliations:** 1 Department of Anatomy, Biochemistry, and Physiology, John A. Burns School of Medicine, University of Hawaii at Manoa, Honolulu, Hawaii, United States of America; 2 Department of Medicine, John A. Burns School of Medicine, University of Hawaii, Honolulu, Hawaii, United States of America; 3 Department of Medicine, Medical University of South Carolina, Charleston, South Carolina, United States of America; 4 Department of Medicine, Ralph H. Johnson Veterans Affairs Medical Center, Charleston, South Carolina, United States of America; The University of Manchester, UNITED KINGDOM

## Abstract

Most cases of congenital obstructive nephropathy are the result of ureteropelvic junction obstructions, and despite their high prevalence, we have a poor understanding of their etiology and scarcity of genetic models. The eight-protein exocyst complex regulates polarized exocytosis of intracellular vesicles in a large variety of cell types. Here we report generation of a conditional knockout mouse for *Sec10*, a central component of the exocyst, which is the first conditional allele for any exocyst gene. Inactivation of *Sec10* in ureteric bud-derived cells using *Ksp1*.*3-Cre* mice resulted in severe bilateral hydronephrosis and complete anuria in newborns, with death occurring 6–14 hours after birth. *Sec10^FL/FL^;Ksp-Cre* embryos developed ureteropelvic junction obstructions between E17.5 and E18.5 as a result of degeneration of the urothelium and subsequent overgrowth by surrounding mesenchymal cells. The urothelial cell layer that lines the urinary tract must maintain a hydrophobic luminal barrier again urine while remaining highly stretchable. This barrier is largely established by production of uroplakin proteins that are transported to the apical surface to establish large plaques. By E16.5, *Sec10^FL/FL^;Ksp-Cre* ureter and pelvic urothelium showed decreased uroplakin-3 protein at the luminal surface, and complete absence of uroplakin-3 by E17.5. Affected urothelium at the UPJ showed irregular barriers that exposed the smooth muscle layer to urine, suggesting this may trigger the surrounding mesenchymal cells to overgrow the lumen. Findings from this novel mouse model show Sec10 is critical for the development of the urothelium in ureters, and provides experimental evidence that failure of this urothelial barrier may contribute to human congenital urinary tract obstructions.

## Introduction

Obstruction of the urinary tract during fetal development causes congenital obstructive nephropathy (CON), the most common cause of chronic kidney disease (CKD) and end stage renal disease (ESRD) in children, and the basis of 16.1% of all pediatric transplantations in the United States [1–3]. This blockage leads to hydronephrosis, a swelling of the kidney due to accumulated urine in the renal pelvis, which is detected by prenatal ultrasound in 1:100 to 1:500 pregnancies [4–6]. The most common cause of CON and infant hydronephrosis is ureteropelvic junction obstruction (UPJ obstruction), where a blockage in urine flow is located at the site where the renal pelvis transitions into the ureter [7–9]. The renal damage from UPJ obstruction is highly variable, ranging from no discernable effects to complete renal atrophy, which may reflect the degree of stenosis. This unpredictability of UPJ obstructions requires constant monitoring, and makes it difficult to decide when surgical intervention is warranted [6,9,10]. Despite the high prevalence and burden of congenital UPJ obstructions, we have a very poor understanding of their etiology.

The mammalian ureter arises from the ureteric bud, an epithelial tubule that sprouts from the nephric duct in response to secreted signals from the metanephric mesenchyme (MM) [11]. While the tip of the ureteric bud grows into the MM and begins to branch into an arborized network of tubules, the stalk of the ureteric bud lengthens and moves down the nephric duct to attach to the urogenital sinus. As the caudal portion of the urogenital sinus differentiates into the bladder, the stalk of the ureteric bud continues to elongate to allow the metanephric kidneys to ascend. The growing ureter also releases paracrine signaling factors to the surrounding mesenchyme to induce differentiation of smooth muscle cells, which will provide strength, elasticity, and peristalsis. Recent advances have shed light on the differentiation of the urothelial cell layers in the bladder [12,13], although this process in the ureter has not been as well studied. By E15.5 in mice, urothelial cells seem to appear in the ureters and by E16.5, the ureter urothelium is multilayered and is resistant to the toxicity of the urine flowing from the kidney [14,15].

Comprised of basal, intermediate and superficial (or umbrella) cell layers, the adult urothelium lines the renal pelvis, ureters, bladder, upper urethra, and prostate ducts [16]. The primary biological role of the urothelium is to provide a highly stretchable, but fluid impermeable, barrier to prevent urine from leaking into the interstitial space. For this purpose, the superficial cells produce large amounts of transmembrane uroplakin proteins that are transported to the luminal surface and form large plaques [16–18].

In eukaryotes, the secretory pathway is essential for crucial cell functions such as delivery of secreted and membrane-bound proteins and the establishment of cell polarity. From the trans-Golgi network, polarized proteins in vesicular carriers are sorted to appropriate sites on the plasma membrane for fusion, and for some locales, this process is mediated by the exocyst complex [19]. Conserved from yeast to humans, the exocyst is comprised of eight proteins and its trafficking activity can be regulated by small GTPases [20,21] or phosphorylation [22–24]. Sec10 is a central subunit of the exocyst complex that connects Sec15, which directly binds Rab GTPases on the intracellular vesicles, to the rest of the exocyst complex in contact with the plasma membrane [25]. Although the exocyst has a wide range of reported functions in various cell types, we are still discovering important roles in polarized epithelia. In cell culture models, we have shown *Sec10* regulates lumen formation in cyst and tubule morphogenesis [26,27], and primary cilia assembly and signaling [27–29]. We previously showed that overexpression of *Sec10* helps maintain renal epithelial barrier function in response to oxidative injury [30]. However, almost all studies of the exocyst in mammalian biology have been limited to cell culture models because general murine knockout of exocyst genes has proved to be early embryonic lethal [31].

In this study, we report generation of a novel transgenic mouse line with a conditional allele for *Sec10*, the first such conditional allele for any exocyst gene. To look at the role of *Sec10* and the exocyst in genitourinary development, we crossed the *floxed-Sec10* mice with the *Ksp1*.*3-Cre* strain, which expresses *Cre* recombinase in epithelial cells derived from the ureteric bud. Surprisingly, this targeted inactivation of the *Sec10* gene resulted in consistent bilateral UPJ obstructions in the upper ureter, with severe hydronephrosis, anuria, and neonatal lethality due to heart failure. The mutant ureters displayed deterioration of the urothelial barrier and rampant overgrowth of surrounding mesenchymal cells to completely fill the ureter lumen at the UPJ. These data suggest that *Sec10* and the exocyst are critical for establishment and maintenance of the urothelial barrier in the ureter. Because the primary cause of the obstructive nephropathy in our mutant mice is a defect in the urothelial cells, we propose the *Sec10* ^*FL/FL*^;*Ksp-Cre* mouse as a unique genetic model for human prenatal UPJ obstructions.

## Results

### Inactivation of Sec10 during kidney and ureter development results in severe hydronephrosis and neonatal lethality

**Fig 1:**
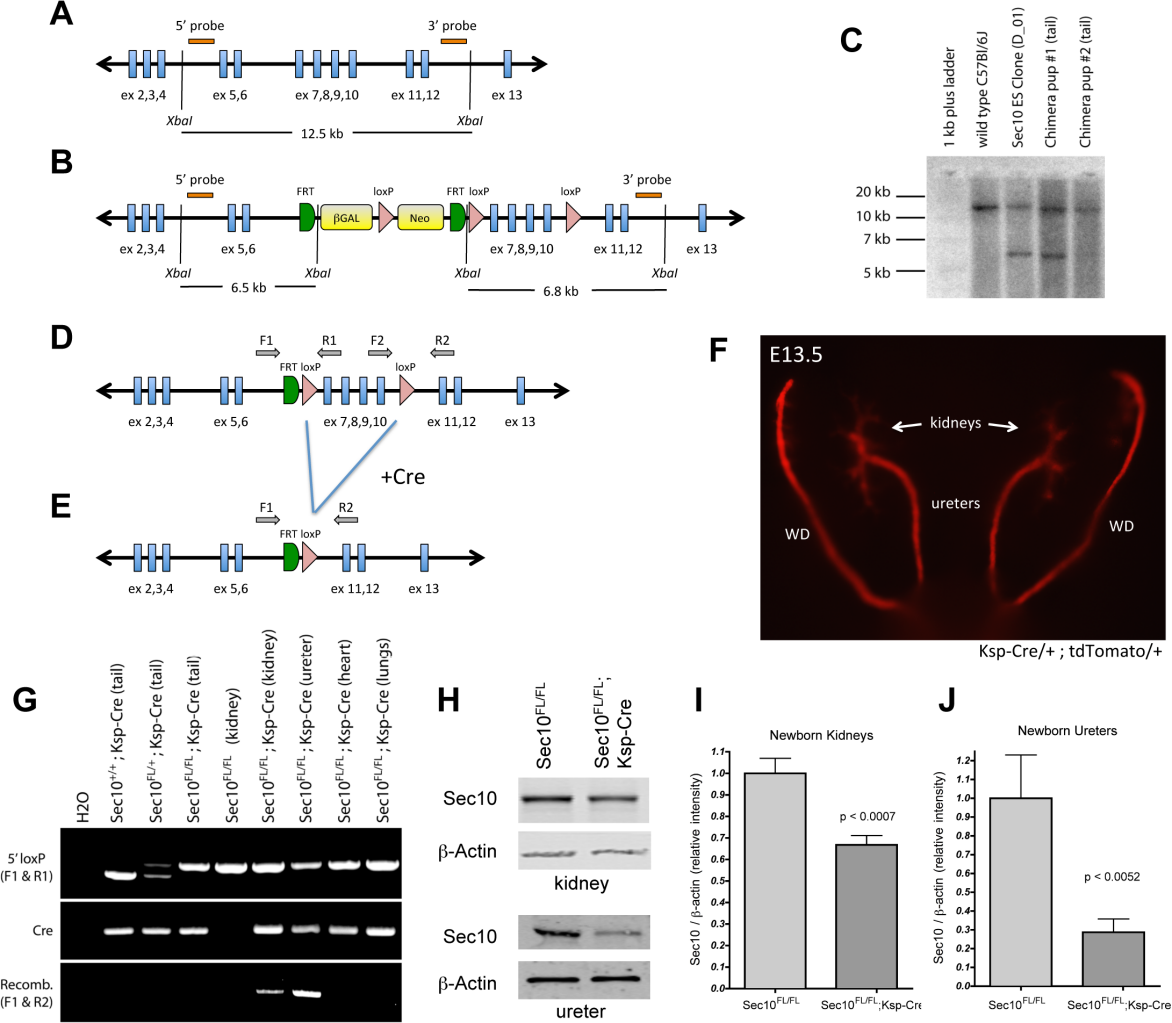
Generation of the *floxed-Sec10* mouse strain and Sec10 conditional knockout mice. (A, B) Shown is a schematic of the murine *Sec10* gene before (A) and after (B) recombination with the *Sec10* conditional targeting vector. Digestion with *XbaI* restriction enzyme yields a 12.5 kb DNA fragment containing exons 5–12 in wild type animals, but the targeting vector introduced new *XbaI* sites to yield smaller fragments. (C) Southern blotting of genomic DNA digested with *XbaI* from wild type C57Bl/6J mice, the injected Sec10 ES clone, and chimeric pups demonstrated homologous recombination with the targeting vector. (D) The final *floxed-Sec10* strain was created by mating mice with germline transmission of the *Sec10* targeting vector with *FLPe* mice to remove the large Neomycin cassette. (E) Upon exposure to Cre recombinase, exons 7–10 were deleted. (F) *Ksp-Cre* mice were crossed with a *tdTomato* reporter mouse strain, and Cre activity was confirmed to be specific to epithelium of ureters, Wolffian ducts (WD), and the collecting system of the kidney as previously reported [35–37]. Shown is the genitourinary system of E13.5 *Ksp-Cre/+;tdTomato/+* embryos, with red fluorescence confirming strong activation of Cre recombinase even at this early stage. (G) PCRs from genomic DNA of various tissues were able to genotype *floxed-Sec10* alleles (upper gel), confirm *Cre* transgenes in our strains (middle gel), and detect deletion of exons 7–10 specifically in *Cre*-expressing tissues (lower gel). Positions of primers used in F are shown in D and E. (H) In *Sec10* ^*FL/FL*^;*Ksp-Cre* mice, Western blotting showed reduced Sec10 protein in whole kidney lysates and isolated ureters, compared with *Sec10* ^*FL/FL*^ littermate controls. (I, J) Western band intensities were measured and Sec10 protein levels were normalized against β-actin and compared via student t-tests (n = 5 for each group, shown are means ± SD).

In order to study the consequences of *in vivo* disruption of Sec10 and the exocyst, we generated a floxed-*Sec10* (*Sec10* ^*FL*^) mouse strain that could be conditionally inactivated when crossed to Cre recombinase-expressing mice. We obtained embryonic stem cell (ES) clones with homologous recombination with a *Sec10* targeting vector from the EUCOMM consortium [32], which contained *loxP* sites flanking exons 7–10 of the *Sec10* gene (Fig 1A and 1B). Recombination via Cre was predicted to delete amino acids 188–312 (of 708 total), with a frameshift and premature stop codon. From 90 blastocysts injected with these ES cells, eight chimeric pups were born, and germ line transmission was verified with both Southern blot analysis and PCR (Fig 1C and 1F). Mating these germline mice with *FLPe* mice [33] removed the Neomycin cassette to produce the final *Sec10* ^*FL*^ strain (Fig 1D). A previous attempt to knock out the exocyst subunit *Sec8* in mice resulted in very early embryonic lethality, during gastrulation [31]. To verify our floxed allele with a global knockout of *Sec10*, we crossed *Sec10* ^*FL/FL*^ mice with *Sec10* ^*FL/+*^;*CMV-Cre/+* mice, which expresses *Cre* in all cells, including germ cells [34]. Despite a theoretical 25% Mendelian inheritance, no *Sec10* ^*FL/FL*^;*CMV-Cre* pups were born alive, and we failed to find any *Sec10* ^*FL/FL*^;*CMV-Cre/+* embryos even as early as E8.5 (n = 76 embryos from 8 pregnancies).

For specific inactivation of the *Sec10* gene in the kidney and ureter epithelium, we crossed the *Sec10* ^*FL*^ mice to the *Ksp1*.*3-Cre* strain (*Ksp-Cre*), which express *Cre* in the branching ureteric buds and all the epithelial cells derived from the ureteric buds. This includes the distal nephron epithelial cells and urothelium of the renal pelvis and upper ureter [35–37]. We verified *Cre* activity in targeted tissues with a *Rosa26-tdTomato Cre*-reporter mouse strain, which reveals Cre recombinase activity through induced expression of red fluorescent protein *tdTomato* [38]. Crossing the *Ksp-Cre* strain with *Rosa26-tdTomato* reporter strain, we confirmed strong activation of *Cre* by E13.5 in ureters, Wolffian ducts, and the collecting system of the kidneys (pelvis and collecting ducts) (Fig 1F). After crossing the *Sec10* ^*FL*^ and *Ksp-Cre* strains, PCR assays were used to confirm deletion of *Sec10* exons 7–10 only in genomic DNA isolated from newborn kidneys and ureters of *Sec10* ^*FL/FL*^;*Ksp-Cre* mice, and not in tissues such as heart or lungs (Fig 1G). Measuring Sec10 proteins levels by Western blots showed a clear decrease in *Sec10* ^*FL/FL*^;*Ksp-Cre* newborn kidneys and ureters compared to *Sec10* ^*FL/FL*^;*+/+* littermate controls (Fig 1, 1H, 1I and 1J). As expected, the ureters, comprised of mainly two cell types, the *Cre*-expressing urothelial cells and the non-*Cre* expressing surrounding smooth muscle cells, demonstrated a much larger decrease in Sec10 level than the kidneys, which contained many cell types not expressing *Cre*.

**Fig 2:**
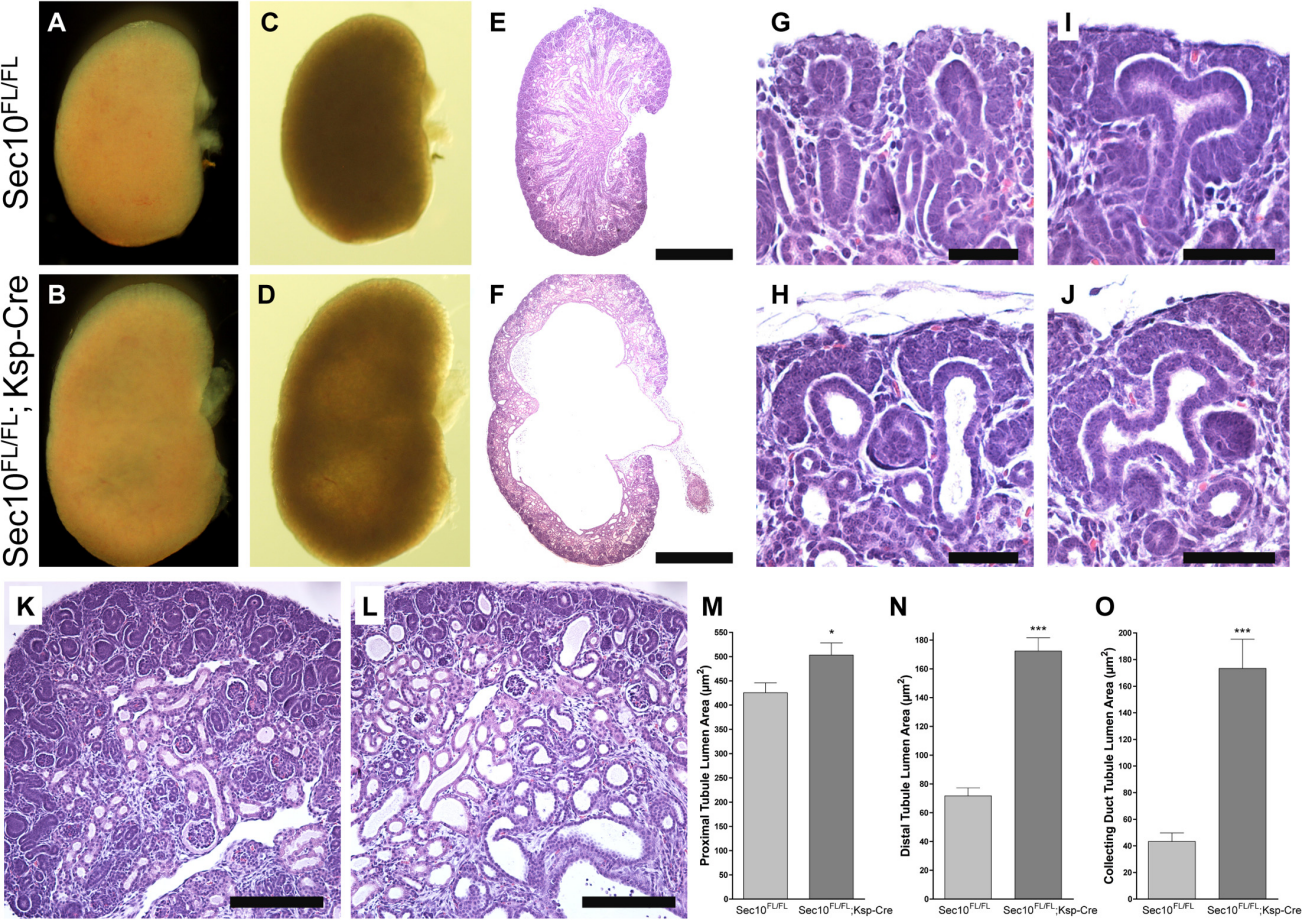
Conditional deletion of Sec10 in upper urinary tract epithelium resulted in severe bilateral hydronephrosis and neonatal lethality. (A–F) Dissected kidneys from E18.5 *Sec10* ^*FL/FL*^;*Ksp-Cre* and *Sec10* ^*FL/FL*^ control littermates demonstrated significant enlargement due to hydronephrosis. Observations from gross dissections (A and B) and backlit images (C and D) were confirmed with H&E stained histology sections (E and F). (G-J) Higher magnification of the cortexes revealed dilations of ureteric bud tips in *Sec10* ^*FL/FL*^;*Ksp-Cre* kidneys that were not observed in *Sec10* ^*FL/FL*^ control kidneys. (K–O) Analysis of dilations of tubular segments was performed from H&E-stained sections, and representative sections are shown (K and L). NIH’s Image J was used to measure luminal surface area of proximal tubules (M), distal tubules (N), and collecting duct segments (O) as identified by tubule morphology. Scale: bar = 1mm (A—F); bar = 25μm (G—J); bar = 100μm (K and L).

We were surprised to observe *Sec10* ^*FL/FL*^;*Ksp-Cre* almost always died 6–14 hours after birth, although a few (<1%) survived past the first day. The longest lived *Sec10* ^*FL/FL*^;*Ksp-Cre* mouse was sacrificed at 6 weeks, and the second oldest mouse survived to 20 days. In all of the dead newborn *Sec10* ^*FL/FL*^;*Ksp-Cre* pups, we observed severe bilateral hydronephrosis, which was never detected in any control littermates (Fig 2A–2D). Histological analysis confirmed these observations (Fig 2E and 2F), and also showed dilations of the tubules all along the nephron, including at the tips of the branching ureteric buds (Fig 2G–2L). We measured and compared luminal surface area of different nephron segments in kidney sections from *Sec10* ^*FL/FL*^;*Ksp-Cre* and control *Sec10* ^*FL/FL*^ newborns, and found the most severe dilations were found in the distal sections of the nephron (Fig 2M–2O). The <1% of *Sec10* ^*FL/FL*^;*Ksp-Cre* mice that survived past birth did not display hydronephrosis, indicating an incomplete penetrance, and that the urinary obstruction contributed to the neonatal death.

### Newborn Sec10^FL/FL^;Ksp-Cre pups have bilateral obstructions in the ureteropelvic junctions which cause complete anuria by birth

**Fig 3:**
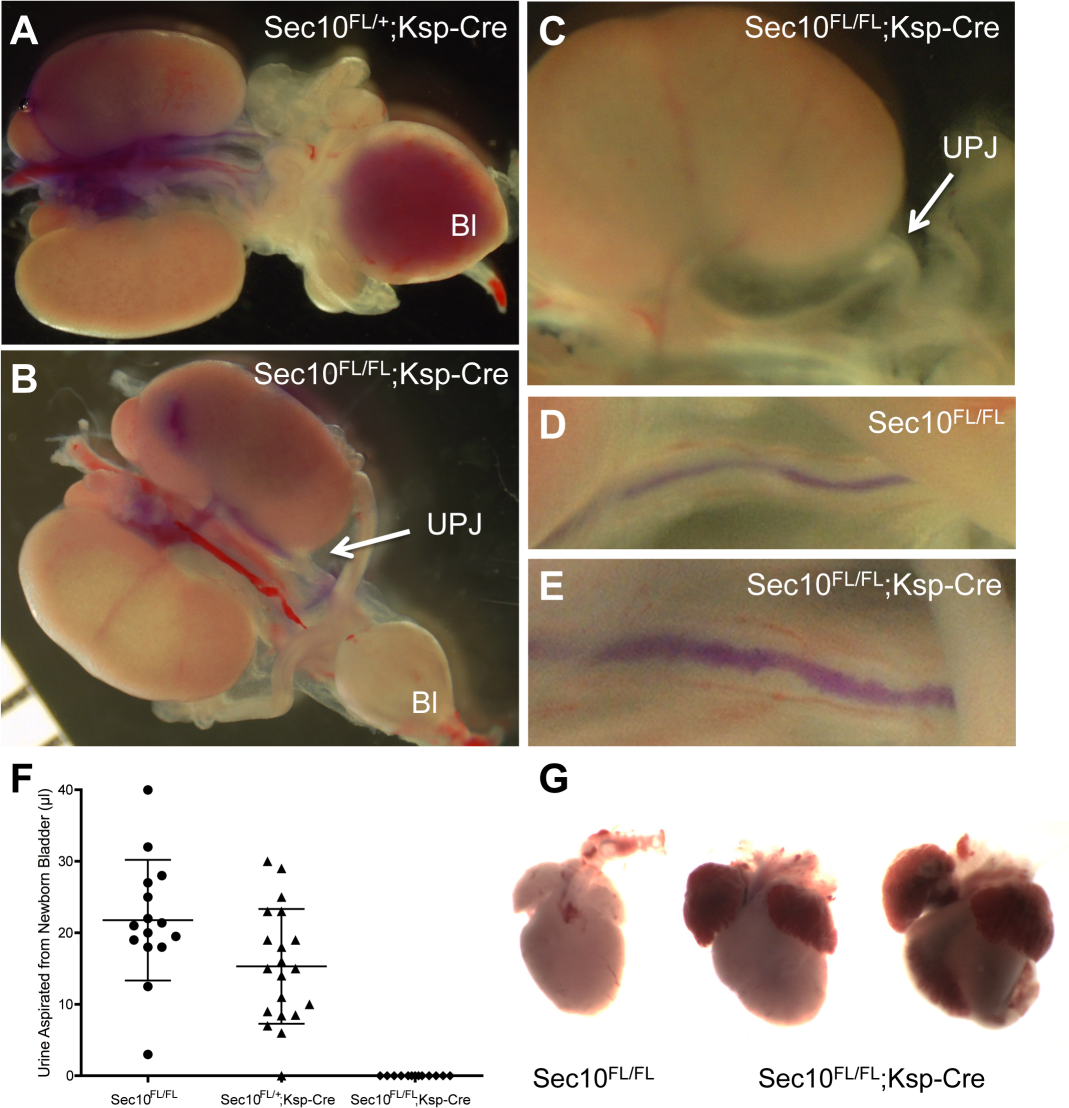
Hydronephrosis in *Sec10* ^*FL/FL*^;*Ksp-Cre* kidneys is due to physical obstructions in ureters at the UPJ, resulting in anuria and heart failure. (A, B) Urinary tracts, including both kidneys, intact ureters, and bladder were removed from mutant *Sec10* ^*FL/FL*^;*Ksp-Cre* mice and littermate controls (*Sec10* ^*FL/FL*^ and *Sec10* ^*Fl/+*^;*Ksp-Cre*) at E18.5 and P0. Blue dye was injected into the renal pelvis, and in control kidneys the dye migrated down the ureters and accumulated in the bladder as expected (representative E18.5 sample shown in A). In every *Sec10* ^*FL/FL*^;*Ksp-Cre* kidney with hydronephrosis tested at E18.5 and P0, the dye stopped at the UPJ (arrow, representative E18.5 sample shown in B). (C) Occasionally in the dissected mutant newborn kidneys with hydronephrosis, a microscopic examination revealed a deposit of white debris within the ureter above the UPJ region, also suggesting a physical blockage of the ureter lumen (arrow, C). (D, E) In one of the few newborn *Sec10* ^*FL/FL*^;*Ksp-Cre* kidneys that did not show hydronephrosis, dye injections traveled to the bladder, but revealed a visibly abnormal ureter lumen with rough irregular edges (E) not observed in controls (D). (F) Aspirations from bladders of newborn pups confirmed that no urine was present in the bladders of *Sec10* ^*FL/FL*^;*Ksp-Cre* pups with bilateral hydronephrosis, compared with a normal distribution found in littermate controls (shown are means ± SD). (G) All newborn *Sec10* ^*FL/FL*^;*Ksp-Cre* pups with bilateral obstructions and hydronephrosis died 6–14 hours after birth, with necropsies revealing heart wall distension and cardiac hemorrhaging. Shown are two representative *Sec10* ^*FL/FL*^;*Ksp-Cre* hearts (right) dissected immediately after death (12-hours post-birth), compared to an age-matched littermate control heart (left).

Hydronephrosis can arise due to physical obstruction of the urinary tract, but also can indicate a loss of smooth muscle strength surrounding ureters or the bladder. To test for urinary obstructions in newborn and E18.5 *Sec10* ^*FL/FL*^;*Ksp-Cre* mice, we used a pulled-glass micropipette to inject blue dye into the renal pelvic area of dissected kidneys. In control kidneys (*Sec10* ^*FL/FL*,^ and *Sec10* ^*FL/+*^;*Ksp-Cre)* without hydronephrosis (n = 41), we could easily observe the dye traveling down the length of the ureter and accumulating in the bladder (Fig 3A). In all *Sec10* ^*FL/FL*^;*Ksp-Cre* kidneys displaying hydronephrosis (n = 14), dye migration would stop at the ureteropelvic junction (UPJ) adjacent to the caudal pole of the kidneys (Fig 3B). In some *Sec10* ^*FL/FL*^;*Ksp-Cre* ureters, microscopic inspection revealed visible deposits of white debris at the UPJ, also suggesting the presence of a physical obstruction (Fig 3C). We also performed these dye injections in one *Sec10* ^*FL/FL*^;*Ksp-Cre* kidney without hydronephrosis, and although the dye traveled down to the bladder, the ureter had visibly abnormal lumens with rough irregular edges, compared with normal controls (Fig 3D and 3E). To measure the degree of obstruction in *Sec10* ^*FL/FL*^;*Ksp-Cre* ureters, we aspirated urine from bladders of 1–4 hour-old pups and compared the collected volume. No *Sec10* ^*FL/FL*^;*Ksp-Cre* pup with hydronephrosis had any detectable amount of urine in the bladder, as opposed to a normal appearing distribution of urine collected from littermates (Fig 3F). Necropsies of *Sec10* ^*FL/FL*^;*Ksp-Cre* pups that died between 6–14 hours after birth revealed gross distension of the hearts with hemorrhaging, which was never observed in control littermates (Fig 3G). We confirmed previous reports that the *Ksp-Cre* mice show no *Cre* activity in the heart or lung tissue by PCR assay (Fig 1G), and by confirming lack of tdTomato fluorescence in heart tissues of *Ksp-Cre;tdTomato* mice (data not shown). Therefore, it is unlikely this cardiac phenotype is due to a congenital heart malformation caused by Sec10 knockout in heart tissue. We also analyzed the weight of lungs in newborn pups, and alveolar surface area, since urinary obstructions are known to influence the volume and content of amniotic fluid *in utero*, but found no differences between mutant and control pups (data not shown).

**Fig 4:**
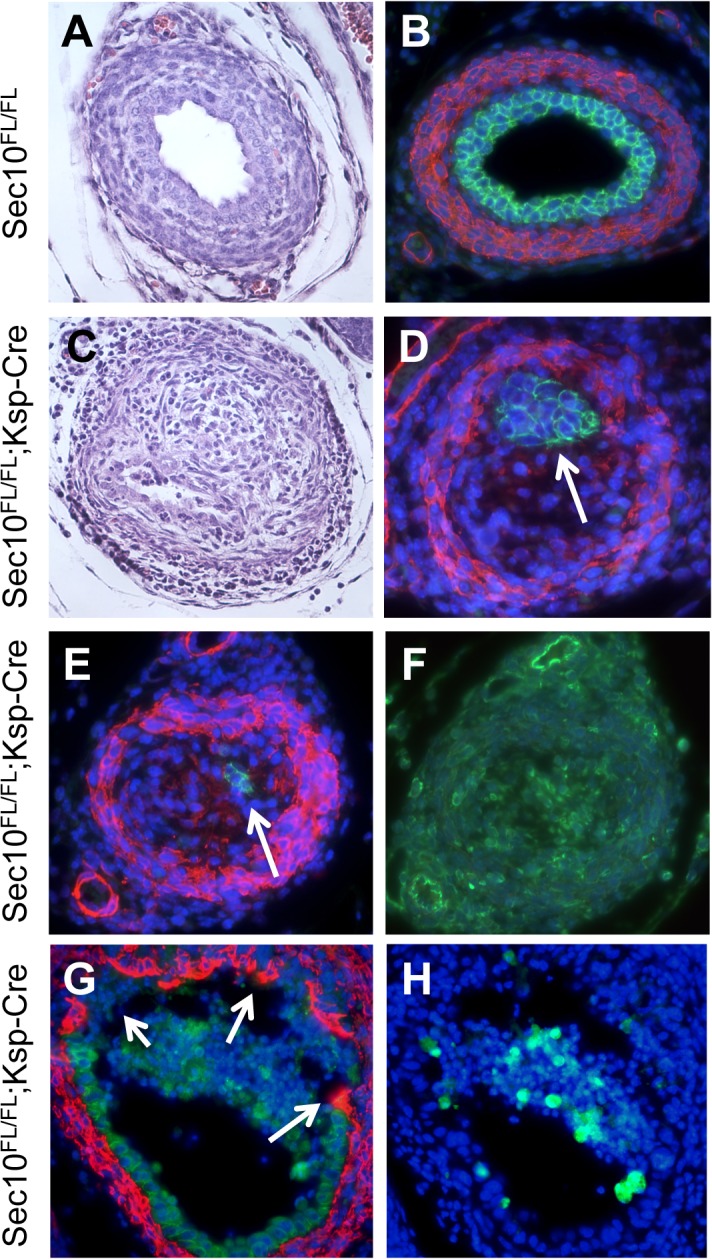
UPJ obstructions in *Sec10* ^*FL/FL*^;*Ksp-Cre* ureters arise from overgrowth of surrounding mesenchymal cells and disappearance of urothelial cells and lumen. (A–F) Histological analysis was performed on newborn *Sec10* ^*FL/FL*^;*Ksp-Cre* ureters and compared with non-obstructed littermate controls. (A, C) At the UPJ region in newborn mice, H&E staining revealed the complete disappearance of lumen in *Sec10* ^*FL/FL*^;*Ksp-Cre* ureters due to overgrowth of cells with a mesenchymal morphology. (B) This was confirmed by immunostaining for E-cadherin (green) and SMA (red), which in control ureters showed a normal cellular organization. (D, E) In *Sec10* ^*FL/FL*^;*Ksp-Cre* ureters, at the UPJ obstruction we found the urothelial cells had greatly reduced in number (arrows), the lumens had completely disappeared, and the overgrown cell population was SMA-positive, E-cadherin-negative (samples from 2 different newborns shown in D and E). (F) In a serial section of the UPJ obstruction shown in E, vimentin immunostaining (green) confirmed cells overpopulating the lumen were mesenchymal, not epithelial. (G) In sections of a rare partially obstructed *Sec10* ^*FL/FL*^;*Ksp-Cre* ureter, E-cadherin (green) and SMA (red) immunostaining revealed damaged urothelium pulling away from the ureter walls, exposing underlying mesenchymal cells to urine (arrows). (H) Immunostaining for activated caspase-3 (green) in a serial section demonstrated many of the detached urothelial cells were apoptotic. Nuclei were counterstained with DAPI.

Using histological methods, we analyzed the morphology of obstructed ureters from newborn *Sec10* ^*FL/FL*^;*Ksp-Cre*, as compared with control littermates. H&E-stained sections of the actual UPJ obstructions in newborn *Sec10* ^*FL/FL*^;*Ksp-Cre* ureters revealed the complete absence of lumens at the blockage, with rampant overgrowth of mesenchymal-shaped cells (Fig 4A and 4C). This was confirmed by immunostaining for E-cadherin and smooth muscle actin (SMA), which showed that in the obstructed segments, few, if any, cells stained positive for E-cadherin (green, Fig 4B and 4D and 4E). Most of the overgrown cells stained positive for SMA (red, Fig 4D and 4E) and vimentin (green, Fig 4F), indicating they may be either smooth muscle cells from the surrounding ureter wall, or myofibroblasts commonly seen in damaged or fibrotic tissues. Occasionally, we observed newborn *Sec10* ^*FL/FL*^;*Ksp-Cre* ureters that were nearly-complete UPJ obstructions, and in one such sample, immunostaining revealed urothelial cells pulling away from the ureteral wall exposing the underlying mesenchymal cells to the urinary lumen (Fig 4G). From staining a serial section of this partial obstruction for active caspase-3, we see the detached urothelial cells were largely apoptotic, but most of the urothelial cells that remained attached were negative for caspase activation (Fig 4H).

Cleaved-caspase-3 immunostaining of *Sec10* ^*FL/FL*^;*Ksp-Cre* ureters from earlier stages (E13.5-E16.5), did not detect any apoptotic epithelial cells, and similar analysis of Sec10-knockout collecting duct epithelia also revealed almost no apoptotic cells. Since Cre activity was clearly evident in targeted epithelial at least by E13.5 (Fig 1F), this suggests Sec10 deletion in urothelial cells does not directly cause cell apoptosis. Rather, the detected urothelial apoptosis later in gestation is most likely a consequence of a disrupted mechanism in ureter development.

### UPJ obstructions develop prenatally in Sec10^FL/FL^;Ksp-Cre embryos between E16.5 and E18.5 and is associated with a disappearance of uroplakins

**Fig 5:**
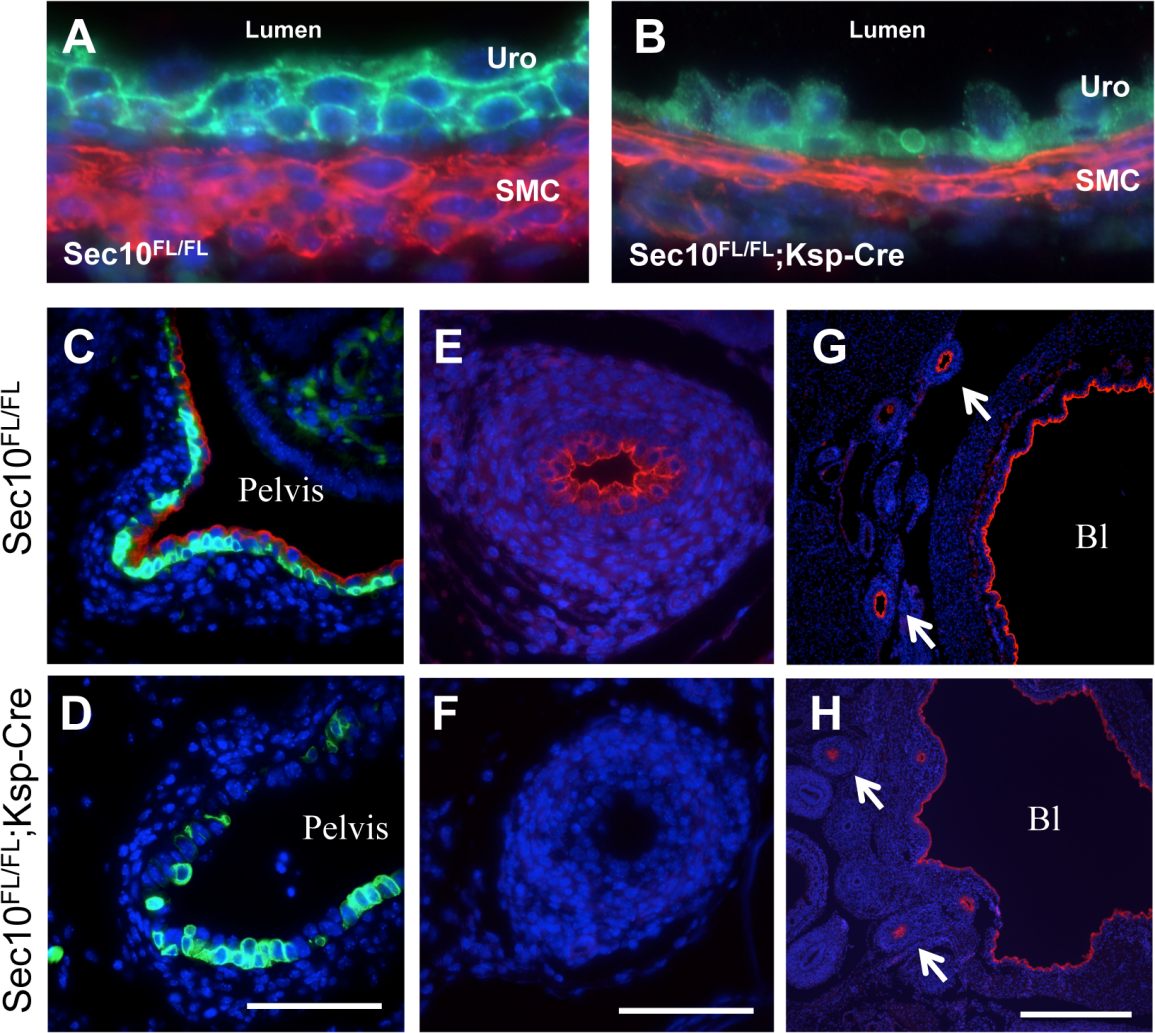
Urothelial cells with Sec10 inactivation displayed an absence of uroplakin-3 at the luminal surface. (A,B) Immunostaining for E-cadherin (green) and SMA (red) in *Sec10* ^*FL/FL*^;*Ksp-Cre* newborn ureters immediately above the blockage (B) revealed urothelial cells that were no longer multilayered, with smaller size and poor cell-cell contact, compared with littermate controls (A). (C,D) Immunostaining for uroplakin-3 (red) and cytokeratin-5 (green) revealed that urothelial cells in the renal pelvis of *Sec10* ^*FL/FL*^;*Ksp-Cre* mice completely lacked uroplakin-3 protein. (E,F) Immunostaining caudal to the UPJ obstruction in newborn *Sec10* ^*FL/FL*^;*Ksp-Cre* ureters confirmed the absence of uroplakin-3 (red) in *Sec10* ^*FL/FL*^;*Ksp-Cre* samples, but not in controls. (G,H) There was no change in uroplakin-3 (red) in the most distal segments of the ureters (arrows) or in the bladder (Bl) of *Sec10* ^*FL/FL*^;*Ksp-Cre* mice. Scale: bar = 0.1mm (B—F); bar = 0.5mm (G and H). Nuclei were counterstained with DAPI.

Above the UPJ blockage, the urothelium of *Sec10* ^*FL/FL*^;*Ksp-Cre* mice had completely lost the normal multilayered formation and was comprised of a single layer of unusually rounded epithelial cells (Fig 5A and 5B). This was accompanied by a thinning smooth muscle cell layer, which appeared abnormally stretched. This urothelial cell phenotype above the blockage has been noted in other models of UPJ obstruction, including surgical models [39,40], and so it may be that this change is secondary to the increased pressure and distension of the renal pelvis and hydroureter. Upon further analysis, we found a complete absence of uroplakin-3 in upper ureters and renal pelvis of newborn *Sec10* ^*FL/FL*^;*Ksp-Cre* embryos, but normal localization in *Sec10* ^*FL/FL*^ and *Sec10* ^*FL/+*^;*Ksp-Cre* littermates (Fig 5C and 5D). As basal urothelial cells express cytokeratin-5 and superficial urothelial cells do not, immunostaining with cytokeratin-5 demonstrated there were some superficial *Sec10* ^*FL/FL*^;*Ksp-Cre* urothelial cells remaining in the pelvis, but these superficial cells did not have any measurable uroplakin-3 (Fig 5C and 5D). In sections of the ureter immediately below the obstruction in newborn *Sec10* ^*FL/FL*^;*Ksp-Cre* mice, a complete absence of uroplakin-3 was observed (Fig 5E and 5F). However, uroplakin-3 was detected in *Sec10* ^*FL/FL*^;*Ksp-Cre* bladders and lower ureters (Fig 5G and 5H), confirming that the absence of uroplakin-3 was limited to urothelium in the upper ureters and renal pelvis.

**Fig 6:**
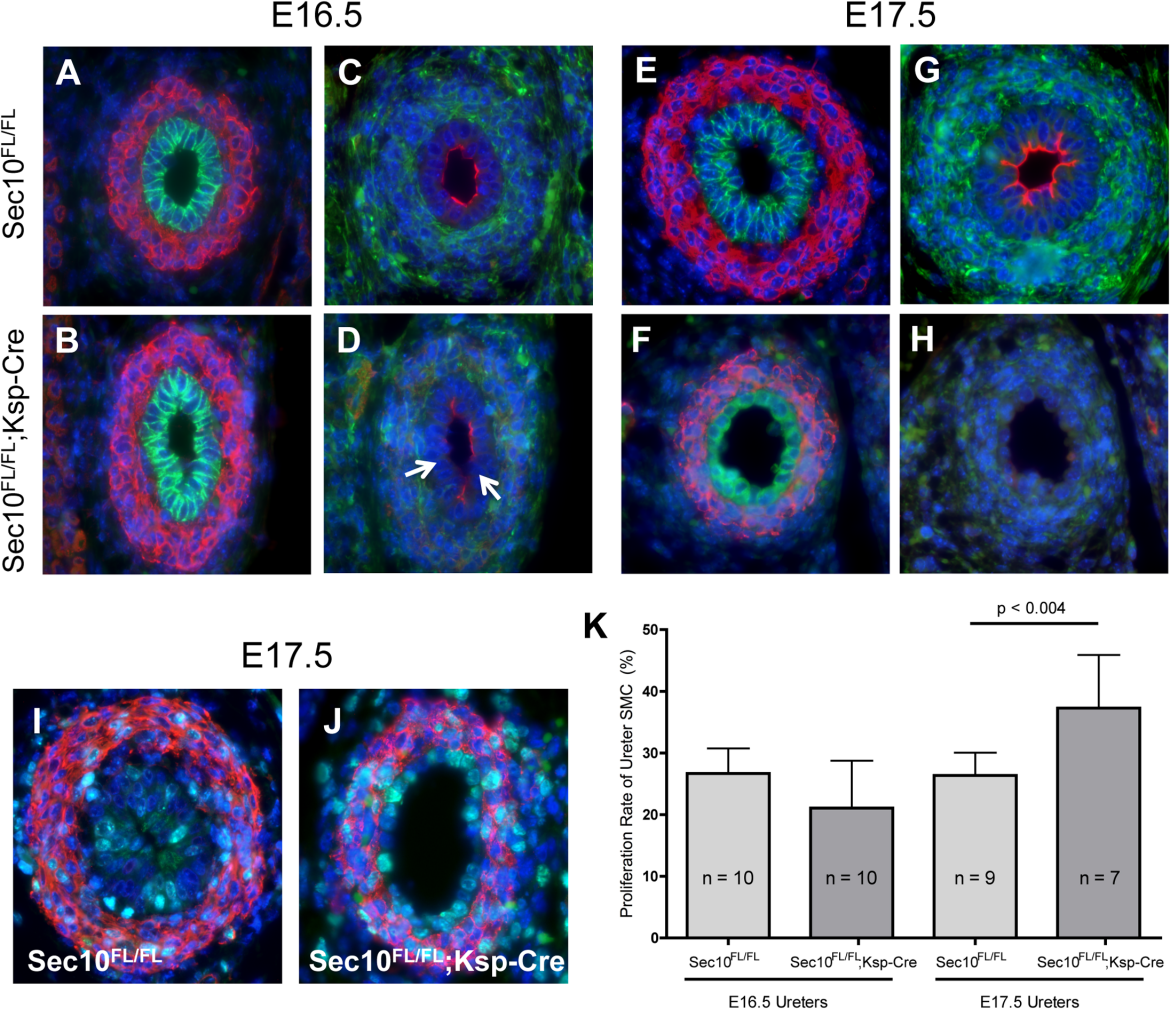
Sec10 is necessary for maintenance of the uroplakin barrier in the developing ureter urothelium. Embryos were collected after timed-matings to evaluate ureters at the UPJ prior to formation of the obstruction, which occurs between E17.5 –E18.5. (A, B) Immunostaining for E-cadherin (green) and SMA (red) at the UPJ in *Sec10* ^*FL/FL*^;*Ksp-Cre* and control E16.5 ureters confirmed initial patency of the mutant UPJs. (C, D) Immunostaining for uroplakin-3 (red) and vimentin to mark the mesenchymal cells (green) revealed that at E16.5, *Sec10* ^*FL/FL*^;*Ksp-Cre* ureters already demonstrated patchy uroplakin-3 localization. Areas of urothelium with noticeably absent uroplakin-3 are marked with arrows (D), while in control ureters, uroplakin-3 was distributed all around the luminal surface (C). (E, F) By E17.5, staining for E-cadherin (green) and SMA (red) revealed that the urothelial layer was visibly abnormal in morphology and consisted of mostly a single layer. (G, H) At E17.5, uroplakin-3 was strongly detectable in all control ureters, but completely absent in *Sec10* ^*FL/FL*^;*Ksp-Cre* ureters around the UPJ. (I, J) To measure proliferation in the smooth muscle layers of developing ureters at the UPJ region, we immunostained for Ki67, which is expressed only in mitotic cells. (K) Proliferation rates were calculated from counting all Ki67^+^;SMA^+^ cells and Ki67^-^;SMA^+^ smooth muscle cells in *Sec10* ^*FL/FL*^;*Ksp-Cre* and control ureters at E16.5 and E17.5. Proliferation rates were compared between mutant and control ureters by student t-tests, with means ± SD and n’s for each group shown. In all images, nuclei were counterstained with DAPI.

Embryos were collected from timed matings to evaluate urothelial changes at the UPJ prior to formation of the full obstruction at E18.5. Immunostaining for E-cadherin and SMA at the UPJ in E16.5 and E17.5 *Sec10* ^*FL/FL*^;*Ksp-Cre* and control ureters confirmed initial patency of the mutant UPJs (Fig 6, 6A, 6B, 6E and 6F). By E17.5, the urothelial layer was visibly abnormal in morphology and consistent of mostly a single layer (Fig 6F). Looking earlier in development, we detected differences in uroplakin-3 levels and distribution in *Sec10* ^*FL/FL*^;*Ksp-Cre* upper ureters as early as E16.5, where uroplakin-3 staining appeared patchy and reduced overall (Fig 6C and 6D). In E17.5 *Sec10* ^*FL/FL*^;*Ksp-Cre* ureters, uroplakin-3 had largely disappeared (Fig 6G and 6H). Since we observed overgrowth of ureter lumen by mesenchymal cells largely occurring by E18.5, we immunostained E16.5 and E17.5 ureter sections at the UPJ region for Ki-67, which is only expressed in mitotic cells (Fig 6I and 6J). We counted cells stained positive for SMA as either Ki67-positive or–negative and statistically compared the percentage of proliferating smooth muscle cells between *Sec10* ^*FL/FL*^;*Ksp-Cre* and control *Sec10* ^*FL/FL*^ ureters. At E16.5, we measure no difference in proliferation rates, but by E17.5, smooth muscle cells in *Sec10* ^*FL/FL*^;*Ksp-Cre* ureters showed significantly higher rates of proliferation (p<0.004; Fig 6K).

## Discussion

We report generation of a conditional murine knockout for the *Sec10* gene, a central subunit of the exocyst complex. This is the first such conditional allele reported for any exocyst gene and should be valuable for studying the exocyst’s role in a large variety of tissues and diseases. The exocyst was initially discovered in yeast by Drs. Peter Novick and Randy Schekman from comprehensive mutagenesis screens for intracellular trafficking genes [41–43], for which Dr. Schekman shared the 2013 Nobel Prize in Physiology or Medicine. Highly conserved from yeast to human, the exocyst has been identified with a rapidly increasing number of biological functions in various mammalian cell types. These recently described roles include GLUT4 trafficking in response to insulin signaling in adipocytes [44], neuronal growth and neuromuscular synapse formation [45,46], cellular invasion of bacteria pathogens and toxins [47–49], tumor invasion and cell migration [50–52], and cellular autophagy [53]. We have been studying the exocyst’s intracellular trafficking to the primary cilia of renal epithelial cells and required an *in vivo* model of exocyst disruption during mammalian kidney development. The one previous knockout of an exocyst component targeted *Sec8* in a general knockout, which resulted in arrested embryonic development at E6.5 [31]. Thus, we embarked to generate a novel murine model for conditional inactivation of *Sec10*, which has shown to be central to the exocyst’s stability and function [27,28,54].

Several conditional alleles of other ciliary proteins have been inactivated with the same *Ksp-Cre* mouse strain used here, with reports of classic polycystic kidneys developing in the first months of life. However, no other conditional knockouts using the *Ksp-Cre* strain to our knowledge has reported a neonatal lethal hydronephrosis or UPJ obstructions. This led us to conclude the phenotype of the *Sec10* ^*FL/FL*^;*Ksp-Cre* embryos is not due to Sec10’s role in primary cilia assembly, and indeed, the mouse urothelium was reported to not be a highly ciliated tissue [55]. Given that the exocyst is a major regulatory complex for polarized exocytosis of intracellular vesicles, we can hypothesize trafficking of uroplakin proteins to the apical membrane of superficial cells, which is the very essence of the hydrophobic barrier of the urothelium, involves the exocyst proteins. There are no previous reports in the literature that have examined the exocyst’s role in urothelial biology. However, Rab8 and Rab11 GTPases, which are known to bind exocyst subunits for targeted secretory vesicle trafficking, have been shown to be important for apical trafficking and “stretch-induced exocytosis” in urothelial superficial cells [56,57].

In our *Sec10* ^*FL/FL*^;*Ksp-Cre* model of prenatal UPJ obstructions, our working hypothesis is that conditional Sec10 knockout in the ureteric bud-derived epithelium leads to disrupted urothelial cell differentiation and failure to establish a hydrophobic barrier in the ureter. This results in urinary damage to the surrounding mesenchymal and smooth muscle cells, and perhaps analogous to arterial restenosis upon damage to the endothelium, this causes over proliferation of these surrounding cell populations. The small diameter of the developing ureter, and relative high hydrostatic pressure at the UPJ through a “bottleneck” effect, results in rapid lumen closure specifically at the UPJ between E17.5 and E18.5. This has been previously proposed by Bartoli at al. as a mechanism for human UPJ obstructions, as damage to the urothelium and overproliferation of mesenchymal cells was been noted by histological analysis of human ureters [58]. Previously studies have generated knockout mice for individual uroplakins, including uroplakin II and IIIa, and reported disruption of the uroplakin plaques in ureters and bladder urothelia [59,60]. However, the urothelial layers in these individual uroplakin knockout mice showed a hyper proliferative phenotype, instead of the apoptotic degeneration observed in the Sec10 mutant mice. These uroplakin knockout mice also survived to adulthood. This highly suggests that Sec10 and the exocyst are critical for urothelial development processes before the final development of the mature uroplakin barrier. Also, in our mouse model, it cannot be ruled out that the knockout of *Sec10* in the renal tubules contributes to or exacerbates the development of the UPJ obstruction and hydronephrosis. Additional studies using other *Cre* mouse strains, including inducible *Cre* variants, will add to our understanding of Sec10 and the exocyst’s role in urinary tract development.

Despite many rodent genetic models with adult onset hydronephrosis, few have presented with prenatal development of congenital obstructive nephropathy [8,40]. One of these is the well-characterized *megabladder* mouse that displays hydronephrosis of variable severity and accompanying renal insufficiency [61,62]. The male megabladder mice live up to 4–6 weeks, and the females live up to 1 year [61], which allows study of renal deterioration. However, this mutant mouse strain has an obstruction in the lower urinary tract with an enormous bladder expansion, while our *Sec10* ^*FL/FL*^;*Ksp-Cre* mice consistently display *in utero* obstructions in the upper ureter. Even fewer defined genetic models of prenatal UPJ obstruction have been discovered, and in those, the targeted knockout has been in the smooth muscle cells surrounding the ureter [63–65]. Uroepithelium-specific knockout of sonic hedgehog did result in hydroureter and secondary hydronephrosis; however, this was non-obstructive in nature and was a result of reduced smooth muscle cell proliferation [66]. Supporting this finding, inhibiting sonic hedgehog signaling components in the mouse ureter mesenchyme led to non-obstructive prenatal hydroureter and hydronephrosis, with variable early lethality [67,68]. Conversely, targeted knockout of members of the TGF-β/BMP signaling pathway in ureter mesenchyme, such as Smad4 and Id2, caused UPJ obstructions likely due to changes in smooth muscle proliferation rates [64,65]. However, the *Sec10* ^*FL/FL*^;*Ksp-Cre* mouse is the first animal model where targeted gene deletion in the urothelial cells of the upper ureter causes prenatal UPJ obstruction, severe hydronephrosis, and neonatal death. Based on the necropsies, we believe the cause of death in bilaterally obstructed pups is heart failure, but substantial cardiorenal and pulmonary physiological assessment on the neonatal pups will be needed to determine the underlying cause of the cardiac dysfunction. Further study of this novel mouse model should allow us to identify new causes of human UPJ obstructions, characterize the progression of congenital obstructive nephropathy disease at a prenatal stage, and perhaps lead to identification of predictive biomarkers for human disease progression and severity.

## Materials and Methods

### Sec10 conditional knockout mice

C57Bl/6J ES clones containing a conditional allele for the *Sec10* (*i*.*e*. *Exoc5*) gene were generated by the trans-NIH Knock-Out Mouse Project (KOMP) and obtained from the KOMP Repository (www.komp.org) [69]. The clone # DEPD00521_3 contains the *Exoc5* ^*tm1a(KOMP)Mbp*^ allele, which was validated by KOMP as having undergone homologous recombination with targeting vector PRPGS00174_A_D07, which contains loxP sites flanking *Sec10* exons 7–10 and *lacZ* and *Neomycin* cassettes flanked by *FRT* recombination sites between exon 6 and 7. Although this vector is designed to cause a general *Sec10* gene knockout, exposure to FLPe recombinase excises the large *lacZ/Neo* cassette and produces a conditional allele (Fig 1). The ES clone was injected into blastocysts (albino C57Bl/6J) by the Engineering Models Resource Core of the HepatoRenal Fibrocystic Diseases Core Center at the University of Alabama at Birmingham, led by Drs. Robert Kesterson and Bradley Yoder, which generated several chimeras with subsequent germline transmission. Genotyping was confirmed using Southern Blotting with probes from both 5’ and 3’ regions after *XbaI* digestion, as well as with genomic DNA PCR (shown in Fig 1). After germline transmission was achieved, heterozygous mice were mated with the *FLPe* recombinase *B6*.*129S4-Gt(ROSA)26Sor* ^*tm1(FLP1)Dym*^ */RainJ* mouse strain [33], obtained from Jackson Laboratories, to remove the *lacZ/Neo* cassette, which was confirmed by PCR and sequencing. We designated the final mouse strain the *floxed-Sec10* line (*Sec10* ^*FL*^). Deletion of *Sec10* exons 7–10 in epithelia derived from the ureteric bud was achieved by mating *Sec10* ^*FL*^ mice with *Ksp1*.*3-Cre* mice [35–37,70,71]. The *Sec10* null allele (*Sec10* ^-^) was generated by mating the *Sec10* ^*FL*^ mice with *CMV-Cre* [34]. The *B6*.*Cg-Gt(ROSA)26Sor* ^*tm9(CAG-tdTomato)Hze*^ */J* reporter mouse strain (kindly provided by Dr. Michelle Tallquist at University of Hawaii) was used to detect *Cre recombinase* activity through *Cre*-activated expression of the *tdTomato* red fluorescent protein [38].

Husbandry and experiments with all mice were approved in advance by the University of Hawaii IACUC, in accordance the American Association of Accreditation of Laboratory Animal Care. Dr. Fogelgren’s IACUC approved protocol is #11–1094, and the University of Hawaii has an Animal Welfare Assurance on file with the Office of Laboratory Animal Welfare (OLAW), assurance number is A3423-01. All animal procedures followed guidelines of the “Guide for the Care and Use of Laboratory Animals” and the “The use of non-human primates in research,” including euthanasia via inhalation of CO_2_. Survival surgeries were not performed in this study.

### Histological Analyses

Tissues were dissected, fixed with 4% formaldehyde, and embedded in paraffin and cut into 10 μm sections. To calculate the luminal surface areas and calculate a cystic index, the largest sagittal section of the kidney containing the cortex, medulla and papilla was stained with H&E. Using the ImageJ software (NIH) [72], the total area of the kidney section was measured, excluding areas around the specimen. Subsequently, as identified by tubule morphology, the total luminal area of different nephron segments within the renal tissue was measured with the same method. Relative luminal areas of the nephron segments were then calculated as a percentage of the total respective lumen area relative to the total area of the kidney section. For immunostaining, tissue sections were deparaffinized, rehydrated in an ethanol gradient, and placed in a pressure cooker for antigen retrieval with citric acid based antigen-unmasking solution (Vector Laboratories H-3300). Sections were blocked with 5% serum, permeabilized with 0.1% Triton X-100, and left in primary antibody overnight at 4°C. Primary antibodies used were anti-SMA (Sigma), anti-E-cadherin, anti-vimentin, anti-Ki67, and anti-cleaved caspase-3 (Cell Signaling Technology), Cytokeratin-5 (Abcam), Cytokeratin-20 (Proteintech Group), and anti-uroplakin III (American Research Products, Inc.). Tissue sections were washed and incubated with DyLight secondary antibodies (Vector Laboratories) for 1 hour at room temperature. Nuclei were stained with DAPI. H&E stained and immunostained sections were analyzed using a fluorescent Olympus BX41 microscope. Image processing, quantification of surface areas, and cell counts were done using ImageJ software (NIH).

### Dye injections to trace urinary tracts

Using timed matings and Theiler staging criteria, litters of E17.5, E18.5, and newborn mice were collected, and their intact urinary tracts removed, including both kidneys, ureters, and attached bladder. Bromophenol blue (1 mg/ml in PBS) was injected into the renal pelvis from a lateral direction using a pulled glass capillary needle connected via catheter tubing to a 1-ml syringe. Dye was injected at a rate of 100 μl/min until it entered the bladder through the ureter, or until the UPJ obstruction became visible. Following injection, imaging was performed using an Olympus SZ-CTV dissecting microscope with an Olympus DP12-2 camera. Tissue was collected from each dissected mouse for genomic DNA isolation and genotyping. Statistical analysis was performed with Prism software (Graphpad).

### Western blotting and real time quantitative PCR

Proteins were isolated from homogenized newborn kidneys and ureters using RIPA buffer with protease inhibitors (Sigma #P8340) and phosphatase inhibitors (Sigma #P5726). Protein lysates were loaded at equal amounts and electrophoresed and blotted using standard methods. Primary antibodies used were anti-Sec10 (Proteintech Group), anti-E-cadherin and anti-Beta-actin (Cell Signaling). Fluorescent secondary antibodies (IRDye) and the LI-COR Odyssey Imager (LI-COR Biosciences) were used for fluorescent detection of proteins, with band intensities quantified using the Licor Image Studio Lite software. RNA was isolated from mouse kidneys and upper ureters using the RNeasy Micro kit (Qiagen), and cDNA was generated with the iScript cDNA Synthesis kit (Bio-rad). Real time quantitative PCR (qPCR) was performed with SYBR Green as previously described [73] using primers against Sec10 and beta-actin (primer sequence available upon request). Amplification and real time measurement of PCR products were performed with CFX96 Real-Time PCR Detection System (Bio-rad).
